# Enhanced Mechanical Properties and Microstructure of Accumulative Roll-Bonded Co/Pb Nanocomposite

**DOI:** 10.3390/nano11051190

**Published:** 2021-04-30

**Authors:** Maryam Karbasi, Eskandar Keshavarz Alamdari, Elahe Amirkhani Dehkordi, Zulfiqar A. Khan, Fariborz Tavangarian

**Affiliations:** 1 Department of Materials Engineering, Isfahan University of Technology, Isfahan 84156-83111, Iran; m_karbasi@cc.iut.ac.ir; 2 Department of Mining and Metallurgical Engineering, Amirkabir University of Technology, Tehran 15875-4413, Iran; alamdari@aut.ac.ir (E.K.A.); ela_amirkhani@yahoo.com (E.A.D.); 3 Department of Design & Engineering, Bournemouth University, Poole, Dorset BH12 5BB, UK; zkhan@bournemouth.ac.uk; 4 Mechanical Engineering Program, School of Science, Engineering and Technology, Pennsylvania State University, Harrisburg, Middletown, PA 17057, USA

**Keywords:** nanostructure, microstructure, composite

## Abstract

Lead composites have been used as anodes in the electrowinning process to produce metals such as copper and zinc. Manufacturing stable lead anodes with appropriate mechanical and chemical properties is required to improve the performance of the electrowinning process. In this study, an accumulative roll bonding (ARB) method was used to fabricate a Co/Pb nanocomposite. Utilizing the ARB method can help us to achieve a uniform structure with enhanced mechanical properties via severe plastic deformation. The results showed that suitable tensile properties were obtained in Pb–0.5%Co–10pass samples. The tensile strength and strain of these samples were 2.51 times higher and 83.7% lower than that of as-cast pure Pb. They also showed creep resistance and hardness up to 1.8 and 2.5 times more than that of as-cast pure Pb. The ARB technique uniformly distributed Co particles in the Pb matrix. The enhanced strength of Pb samples was observed in the composite including grain sizes of less than 50 nm as a result of hindering the recovery phenomenon. The particle size of the Co distributed in the Pb matrix was 353 ± 259 nm. Compared to conventional methods, the ARB process improved the mechanical properties of Co/Pb composites and can open a new horizon to fabricating this composite in metal industries.

## 1. Introduction

The electrowinning process is one of the essential methods in producing metals such as copper and zinc. Electrowinning is the cathodic reduction of a metal from a metallic ion containing an aqueous solution via an electrical current using the electrolysis method [1]. In this technique, the final products such as Zn and Cu are deposited on an Al, Ti, or steel [2] cathode. It is important to make sure that anodic materials are insoluble in a bath since even low solubility of anode materials can contaminate the cathodic metal products.

The properties of anodes must achieve a cathodic metal product of high quality. The properties of anodes have a direct influence on the quality and the cost of products. Generally, anodes should have some specifications such as high electrical conductivity (high energy productivity) and long-term stability (high corrosion and wear resistance, which sustain the initial dimension of anodes in acidic solutions). These properties decrease product contamination, energy and materials consumption, as well as maintenance costs [3].

Lead (Pb) was selected as an electrowinning anode due to its relatively high corrosion resistance in a sulfuric acid bath [4]. However, it has some disadvantages such as low yield strength, tensile strength, hardness, and creep resistance, which decrease anode lifespans and raise the cost of production [5]. Utilizing alloying processes has been suggested in the literature to improve the behavior of lead anodes in electrowinning processes [6]. The preparation of lead alloys (for example, in Pb–Co systems) is accompanied by some challenges, one of which is the immiscibility of most of the elements in lead [7]. Cobalt has been added to lead anodes as an alloy element since 1917 [8]. The addition of cobalt to lead anodes is accompanied by an electro-catalytic improvement. Pb–Co is an immiscible metal composite system. The immiscibility of cobalt in Pb (less than 0.02 wt.%) is accompanied by a microstructural heterogeneity of lead alloyed anodes. However, segregation and heterogeneous distribution of alloying elements during casting can lead to a significant decrease in the reliability and controllability of lead alloyed anodes in industrial performance [8]. Some techniques, such as pre-alloying Co with Sb, Sn, and Bi and then alloying with Pb; cementation of Co in liquid lead chloride salt; melting of Pb and Co followed by fast cooling; pulse electroplating; mechanical alloying; and spraying of Co on the surface of Pb sheets followed by rolling, have been examined by some researchers [7]. However, due to a significant difference in the melting points of Co and Pb (about 1168 °K at the liquid phase), poor mechanical and structural properties of the final products, high production costs, and the complexity of the suggested methods [7], the majority of these techniques cannot be used at industrial scales. 

Accumulative roll bonding (ARB) is an intensive plastic deformation technique that has several advantages over other methods such as cost-effectiveness and a high production rate [9]. This method has been examined in many metals’ systems such as Al [10] and Cu base composites [11], brass [12] and steel alloys [13], and metal matrix composites.

In this study, the accumulative roll bonding (ARB) method was used to fabricate a Co/Pb composite. The ARB-produced Co/Pb anodes were characterized and then composites were produced with as-cast lead anodes, ARB-processed Pb, and Pb–Ag 0.5% composite sheets (as the RSR commercial anode brand).

## 2. Materials and Methods

Lead ingot (99.98 wt.% Pb), from National Iranian Lead and Zinc (NILZ) Co, Zanjan, Iran (Table 1), was used to prepare the composite sheets. Co (Fluka Co 99.93 wt.% Co) powders with particle sizes in the range of 1–3 µm were used in the secondary phase. 

To prepare composite sheets, the surface of two strips measuring 100 mm × 50 mm × 3 mm were conditioned with pure acetone and scratched using a rotating wire brush. After surface treatment, the particles were evenly dispersed between the upper sides of the strips via a vibration dispenser. Then, the strips were stacked over each other and fixed at both ends. The roll bonding process was conducted without any lubrication at a loading capacity of 20 tons and a 50% reduction. For the next step, cold roll bonded strips were sectioned in half, conditioned, and scratched and the powder was dispersed and then fastened at the end of the strip. This procedure was repeated for up to 4 cycles at room temperature (the first step). ARB cycles were repeated without the addition of powder for up to a defined pass for better distribution (the second step). The specifications of the prepared sample are outlined in Table 2. 

Tensile test specimens were manufactured using a wire cutting instrument on rolled strips that were oriented along the rolling direction, according to the ASTM E8M standard [14]. A Hounsfield H50KS testing machine at a speed of 10 mm/min was used for the tensile tests. In order to evaluate the behavior of samples subjected to shear forces, shear punch tests were performed in the normal direction, based on the ASTM E8–98 standard. It must be mentioned that a shear punch fixture with a 3.175 mm diameter flat cylindrical punch and a 3.225 mm diameter receiving hole was used. The micro-hardness of the samples was measured in a rolled plane under a load of 15 g via the Buehler Micro-Met 5105 system. Hardness was measured randomly at 10 different points on the strips for each sample. All data were examined as average values ± standard deviation for *n* = 3. Statistical analysis was performed using GraphPad Prism. The data were analyzed for statistically significant differences with 2-way ANOVA, *p* < 0.05.

Electron microscopy and energy dispersive spectroscopy (SEM-EDS PHILIPS XL 30, Eindhoven, The Netherlands, at an accelerated voltage of 25 kV) were employed to evaluate the microstructure of test samples. Transmission electron microscopy (TEM, PHILIPS CM120, Massachusetts, USA, at 200 kV) was used to examine the structure of the ARB-processed specimens. Microstructural changes in different samples were characterized by X-ray diffraction (XRD, Philips Xpert, Eindhoven, The Netherlands) with Cu Kα radiation (λ = 0.154 nm, 40 kV, and 30 mA) in the 2θ range of 20–90°.

## 3. Results and Discussion

### 3.1. Mechanical Properties

#### 3.1.1. Tensile Test

**Figure 1:**
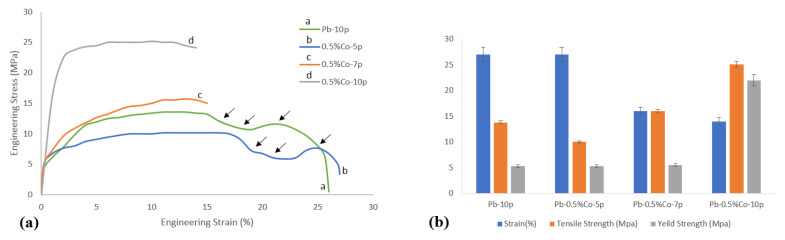
(**a**) Stress–strain curves and (**b**) yield strength, tensile strength, and strain of various composites.

The results of the tensile test are shown in Figure 1. It is evident from the results that, with the increasing number of ARB cycles, strain and tensile strength decreased and increased, respectively. The increase in strength and the decrease in strain were intensified by increasing the number of passes. Pb–0.5%Co–10pass had the highest tensile strength (which was 2.51 times higher than that obtained from the pure Pb strip (Table 3)) and the lowest strain (14% in comparison with 87% for the Pb 0–pass sheet (Table 3)). The increasing strength after 10 ARB cycles can be ascribed to the better Co phase distribution in the matrix, which could be considered one of the advantages of the ARB technique [15]. It has been demonstrated that the Young’s modulus increased (Figure 1b) with the number of ARB cycles. The increase in the Young’s modulus was noticeable at the 10th pass in Co-containing samples. This behavior occurred due to the severe anisotropic plastic deformation (SPD) caused by the ARB process as well as the presence of the secondary phase [16].

Some stress oscillations in the strain–stress curves could be observed in Figure 1a (curves a and b). After 5 passes, in the presence of Co (Figure 1a (curves c and d)), the stress oscillations were omitted (please see the arrows). Stress oscillations occurred as a result of the counteraction between work-hardening and the accelerated recovery phenomenon in the lead structure [17]. The work-hardening behavior could be compensated by structural recovery. Dynamic recovery of the Pb matrix was accelerated due to its very low melting point. This phenomenon could be hindered significantly by uniformly distributing the second phase particles in the matrix and by increasing the ARB passes, resulting in higher SPD work-hardening [15]. Therefore, higher stress was required for further strain during tensile deformation. Subsequently, stress oscillations disappeared at the higher ARB passes. 

**Figure 2:**
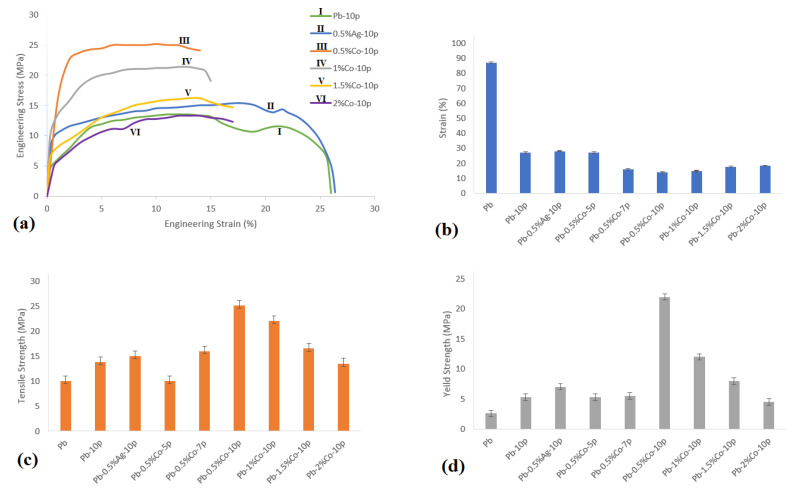
(**a**) Stress–strain curves, (**b**) yield strength, (**c**) tensile strength, and (**d**) strain of various composites.

Figure 2 shows the strain–stress curves of the Pb–Co composite at various Co contents in comparison with the Pb–0pass and Pb–0.5%Ag after 10 ARB passes. The results indicated that strain and strength were optimized in the presence of 0.5 wt.% Co, as compared to the Pb–0.5%Ag–10pass and samples with higher contents of Co. However, specimens with a Co content greater than 0.5 wt.% showed lower tensile strength (see Figure 2a). This can be ascribed to the aggregation of Co particles between ARB-initiated layers, which was accompanied by lowered bond strength [14]. Therefore, the improved tensile properties in ARB-processed Co/Pb composites were optimized to be at 0.5 wt.% Co.

#### 3.1.2. Hardness Test

**Figure 3:**
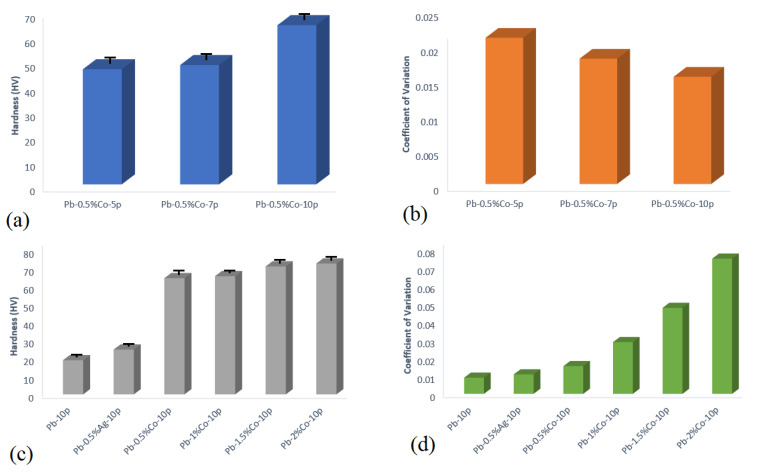
(**a**,**c**) Hardness and (**b**,**d**) coefficient of variation of different samples.

The hardness test was performed to evaluate the surface deformation resistance of anodes. Figure 3 shows the results of the hardness test of Pb–0pass and Pb–0.5%Ag–10pass in comparison with Co/Pb composites at different ARB cycles and Co content. The mean value and the coefficient of variation of the measured data were calculated. The coefficient of variation (Cv) was calculated using the following equation:

Cv = σ/µ(1)

where σ is the standard deviation and µ is the average value. The coefficient of variation is a dimensionless and average independent value that is suitable for data comparison. 

As shown in Figure 3, the hardness of specimens increased (Figure 3a) and Cv decreased (Figure 3b) with increases in the number of ARB cycles (46.5 Vickers in Pb–0.5%Co–5pass samples, compare to 64.3 Vickers in Pb–0.5%Co–10 pass samples). Increasing the hardness up to the maximum amount after the 10th ARB cycle was the result of improved strength as a characteristic of severe plastic deformation techniques. The lower calculated Cv at higher ARB cycles was due to the improved particle distribution between the layers through the ARB process. Additionally, it was observed that the hardness (Figure 3c) and Cv (Figure 3d) of specimens increased by increasing the Co content. It could be due to the increased hardness of Co powder, compared to Pb and Ag (the hardness of silver and lead was 2.5 and 1.5 on the Mohs hardness scale, respectively). The higher coefficient of variation of samples containing cobalt, compared to samples containing silver at similar ARB passes, was because of the difference in hardness of cobalt and silver. At similar ARB pass cycles, the coefficient of variation was increased by increasing the weight percentage of cobalt. This can be ascribed to the reduction in the secondary phase distribution during ARB passes. This behavior was reported by other researchers as well [14,17]. Additionally, it should be mentioned that, in these metal matrix composites, the softer metal (lead) was the matrix and the harder metals (Co and Ag) were the reinforcement. During the hardness test, different locations were examined and the mean value reported was due to the fact that the indenter could land on top of the reinforcement particles and thus report a higher value of hardness.

#### 3.1.3. Shear Punch Test

The results of shear punch tests are shown in Figure S1. In this Figure, three stages can be seen; at the first stage (1), greater force was required for further punch displacement due to the work-hardening phenomenon. At the second stage (2), work hardening and dynamic structural recovery were balanced and an almost constant force was required to continue the shear deformation. In the third stage (3), dynamic structural recovery was enhanced and the required force for shear deformation decreased. As can be seen, a significantly greater force was needed for certain displacement levels in the Pb–0.5%Co–10pass sample. For example, to obtain 0.4 mm displacement in the Pb–0.5%Co–10pass sample, 150 N force was required, but in other samples, less than 80 N was enough for the same displacement. However, up to three times more force was required for 0.5 mm punch displacement in the Pb–0.5%Co–10pass, as compared to the Pb–0pass specimen [18]. 

Figure S2 shows the results of the shear punch creep test (SPCT). In this figure, normalized punch displacement δ (mm/mm) were plotted vs. time (min). δ was calculated based on the following equation:

Δ = h/t(2)

where h is the displacement and t is the specimen thickness. As can be seen, SPCT curves had a similar shape to traditional creep curves, which could be used for creep behavior description [18]. Comparing the curves suggested a lower shear deformation rate in all ARB-processed samples compared to the pure Pb–0pass specimen. Among all the samples, the Pb–0.5%Co–10pass had the lowest deformation rate. The significantly lower creep rate of the Pb–0.5%Co–10pass sample was due to diminished structural recovery and enhanced shear deformation work-hardening. Anisotropic severe plastic deformation as a result of the ARB process as well as the presence of Co (as a harder additive when compared to Ag) could lead to the creep behavior in the Pb–0.5%Co–10pass [18]. 

For shear stress calculation, the following equation could be used [19]:

τ = P/πdt(3)

where τ is the shear tension (MPa), P is the ultimate shear force (N), d is the average punch and matrix diameter (mm), and t is sheet thickness (mm). Figure S3 shows the tensile and shear strengths of different samples. As can be seen, the variations in shear strength were in good agreement with tensile strength due to the formation of a homogenous composite microstructure.

### 3.2. Microstructure Analysis

**Figure 4:**
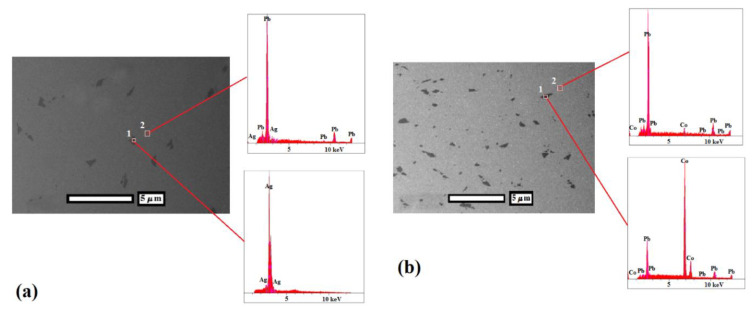
BSE and EDS analyses of (**a**) Ag/Pb and (**b**) Co/Pb composites after 10 ARB passes.

In order to evaluate the distribution of the secondary phase in the Pb matrix, back scattered electron (BSE) micrographs were used, as shown in Figure 4. Two distinct regions could be seen in the BSE micrographs. Energy dispersive spectroscopy (EDS) results showed that dark regions in the BSE micrographs were related to the presence of the secondary phase. As can be seen, finer particles (less than 1 μm) as well as more homogeneous distribution (less than 2 µ apart) were achieved in the Pb–0.5%Co–10pass in comparison to the Pb–0.5%Ag–10pass sample (generally, more than 1.5 μm phase size with particles more than 3 μm apart). This can be ascribed to the lower ductility of cobalt in comparison to Ag, leading to the enhanced work hardening and faster breaking down of Co particles [14]. In both samples, the continuity of the interfaces between the secondary phase and the matrix was desired without any gap as a consequence of super ductility and lubrication characteristics of the Pb matrix. The particle size distribution of Pb–0.5%Co–10pass and Pb–0.5%Ag–10pass samples was 353 ± 259 and 553 ± 286 nm, respectively.

**Figure 5:**
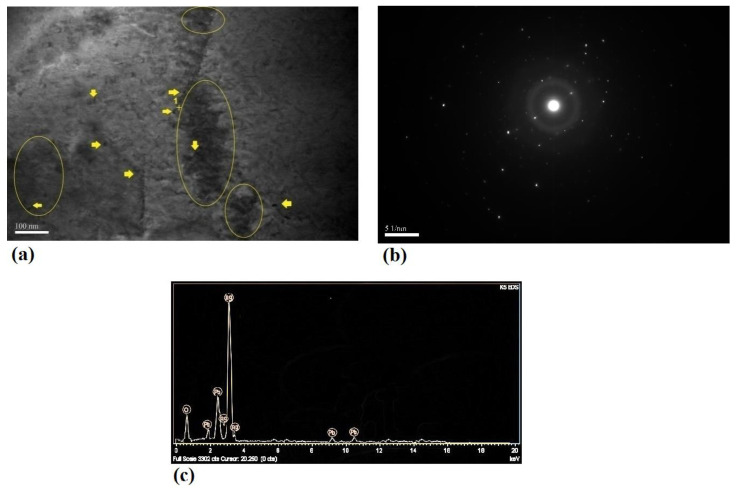
(**a**) Bright field image, (**b**) SAD pattern, and (**c**) EDS result of Pb–0.5%Ag–10pass.

**Figure 6:**
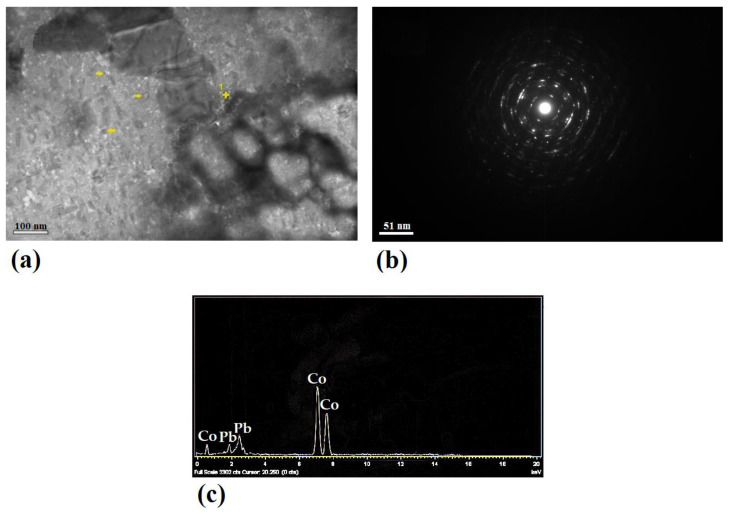
(**a**) Bright field image, (**b**) SAD pattern, and (**c**) EDS result of Pb–0.5%Co–10pass.

**Figure 7:**
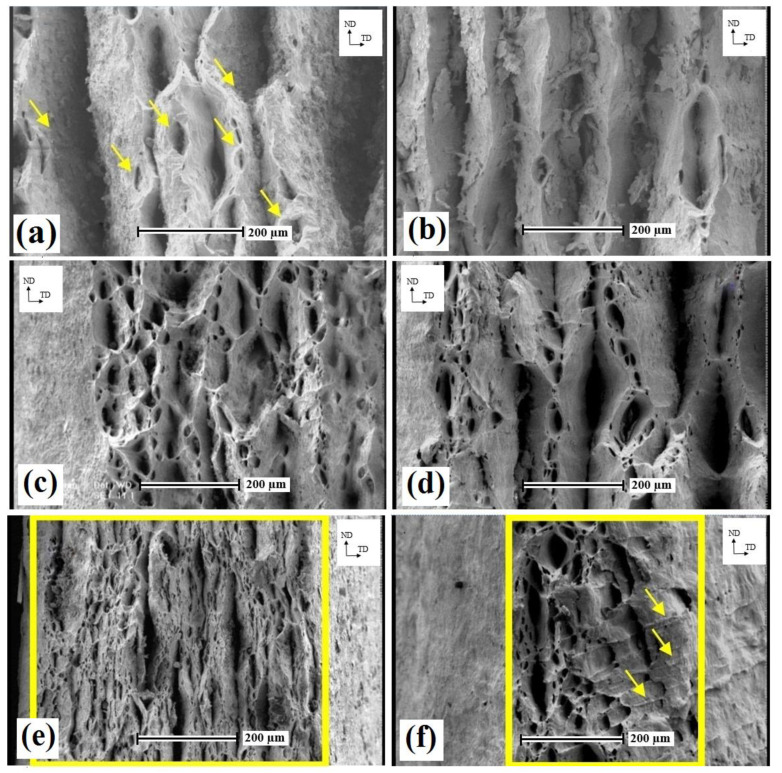
SEM micrographs of the fractured surface of (**a**) Pb–0.5%Co–5pass, (**b**) Pb–0.5%Ag–5pass, (**c**) Pb–0.5%Co–7pass, (**d**) Pb–0.5%Ag–7pass, (**e**) Pb–0.5%Co–10pass, and (**f**) Pb–0.5%Ag–10pass.

Figure 5 and Figure 6 show the bright field, SAD pattern, and EDS of different specimens. A significantly higher dislocation density as well as an enhanced formation of dislocation tangles (outlined zones), cells, and sub-grains was observed in Pb–0.5%Co–10pass compared to the Pb–0.5%Ag–10pass sample. In addition, some dark particles were visible at grain boundaries and dislocation tangles (arrowed). The presence of secondary phase particles with less than 5 nm in size at the arrowed zone was proved by EDS analysis (Figure 5c and Figure 7c). The impact of the more brittle secondary phase (Co in comparison with Ag) on the strengthening phenomena (grain boundaries pinning and dislocation bowing) was noticeable.

As a result, secondary phase particles could inhibit grains’ growth, leading to a strongly pinned microstructure. Pinning of grain boundary was enhanced by increasing the volume fraction of secondary phase particles. The lower density of cobalt (8.9 g/cm^3^) compared to silver (10.5 g/cm^3^) at the same weight percentage was equal to a higher volume fraction and the intensified grain boundaries pinning with diminished grain growth. This mechanism could be clearly supported by comparing the dot-like SAD pattern of the 0.5%Ag–10pass sample (Figure 5c) with the ring-shaped pattern in the 0.5%Co–10pass specimen. Additionally, nano-sized secondary phase particles could act as a dislocation movement barrier leading to dislocation bowing and subsequently microstructure strengthening. In this strengthening mechanism, harder Co particles could be more effective (Co and Ag hardness in the Mohs scale was 5 and 2.5, respectively).

Figure 7 shows the fractured surface of Pb–0.5%Co and Pb–0.5%Ag samples at different numbers of ARB cycles. At the 5th ARB pass (Figure 7a,b), deformed layers could be seen in the fractured surface of the Pb–0.5%Ag sample, but in the Pb–0.5%Co sample, a bonded layer with fine dimples was observed. This behavior was due to the significantly stronger action of the micro-punching mechanism [20] and the higher bond strength of the Pb matrix layer in the presence of harder Co particles in comparison with Ag particles. After the 7th ARB pass (Figure 7c,d), the bonded layers could be seen in Pb-Ag samples. 

At the 10th pass (Figure 7e,f), the ARB-processed samples exhibited a typical ductile fracture, indicating deep equiaxed dimples. In the Pb–0.5%Ag–10pass sample, shear zones (arrowed) could also be observed. These behaviors could be a result of the higher deformation resistance in Pb–Co due to the presence of Co particles. Such resistance could act as a stronger barrier for deformation phenomenon during the tensile test, leading to an enhanced strengthening mechanism and the accelerated work hardening. By increasing the number of ARB cycles, the number of voids was increased and, consequently, the size and the depth of voids were decreased. This can be ascribed to the improved deformation resistance and stronger hindering of dynamic recovery during the tensile deformation of the Pb–Co sample.

**Figure 8:**
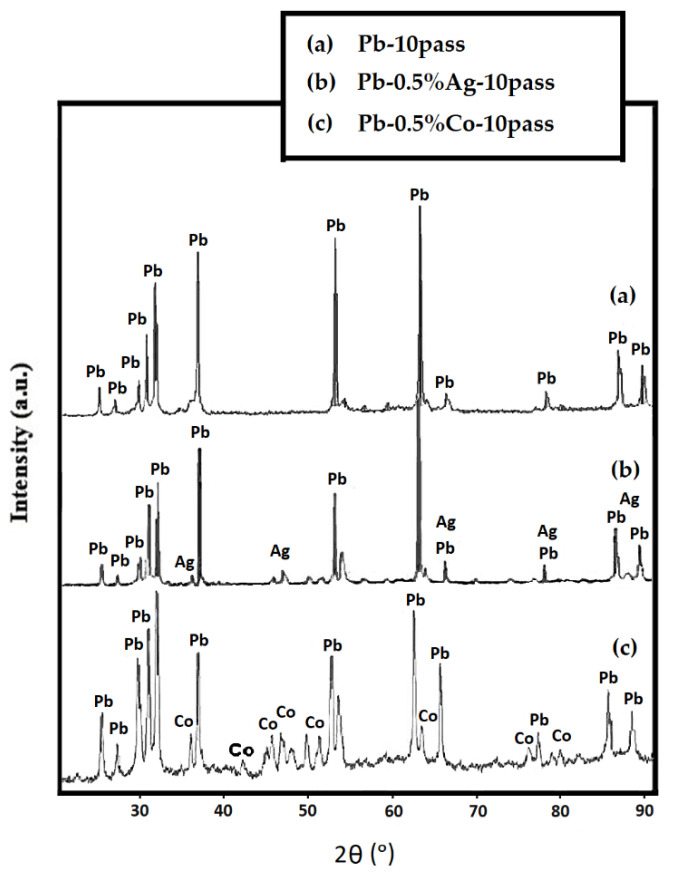
XRD pattern of (**a**) Pb–10pass, (**b**) Pb–0.5%Ag–10pass, and (**c**) Pb–0.5%Co–10pass.

To investigate the microstructure of various samples, X-ray diffraction patterns were obtained. Figure 8 shows the XRD patterns of Pb–10pass, Pb–0.5%Ag–10pass, and Pb–0.5%Co–10pass samples. The XRD peaks of pure Pb and the sample containing Ag showed that the height, intensity, and location of Pb peaks did not change considerably; however, in the presence of Co particles, the height of characteristic peaks of Pb decreased and the width of peaks increased due to the severe plastic deformation that occurred via the ARB process as well as the formation of a fine microstructure. To evaluate the microstructure of the samples, the Rietveld XRD analysis method was used by ExpertPlus software (Table 4). Higher dislocation density and lower sub grain size in the Pb–0.5%Co–10pass was observed due to the diminished recovery phenomenon in the presence of hard and homogenous distributed particles. These results are in good agreement with other results, which are reported in previous sections. 

## 4. Conclusions

From the above study, the following conclusions are drawn:

(1) ARB was used to fabricate nano-structured Co/Pb composite anodes.
(2) The maximum tensile strength was gained in the Pb–0.5%Co–10pass sample (1.5 times compared to Pb–0pass).
(3) Creep resistance increased for the Pb–0.5%Co–10pass up to 1.8 times compared to Pb–0pass.
(4) Up to 2.5 times increased hardness was achieved in Pb–0.5%Co–10pass compared to Pb–0pass.
(5) The ARB process led to an appropriate distribution of Co and Ag secondary phase particles, with particle sizes of 353 ± 259 and 553 ± 286 nm, respectively.

The results of this study showed that the ARB process can enhance the mechanical properties of Co/Pb anodes used in electrowinning processes, which results in lower manufacturing costs of the final products.

## Figures and Tables

**Table 1:** Chemical composition of the Pb ingot.

Content	Pb	Sn	Zn	Ni	Sb	Bi	Ag
wt.%	99.987%	0.0011%	0.0041%	0.0003%	0.0059%	0.0012%	0.0006%

**Table 2:** Designation of different specimens.

Sample Code	Added Powder (wt.%)	Number of ARB Cycles
Pb–0 pass	0	0
Pb–5 pass	0	5
Pb–7 pass	0	7
Pb–10 pass	0	10
Pb–0.5%Ag–10 pass	0.5	10
Pb–0.5%Co–5 pass	0.5	5
Pb–0.5%Co–7 pass	0.5	7
Pb–0.5%Co–10 pass	0.5	10
Pb–1%Co–10 pass	1	10
Pb–1.5%Co–7 pass	1.5	7
Pb–2%Co–7 pass	2	7

**Table 3:** Yield strength, tensile strength, and strain of various samples.

	Increase Yield Strength (Time)	Increase Tensile Strength (Time)	Decrease Strain%
Pb–0pass	---	---	---
Pb–5pass	0.1	0.1	14
Pb–7pass	0.6	0.3	33
Pb–10pass	1.0	0.3	69
Pb–0.5%Ag–5pass	1.2	0.0	34
Pb–0.5%Ag–7pass	1.3	0.3	50
Pb–0.5%Ag–10pass	1.6	0.5	68
Pb–0.5%Co–5pass	1.0	0.0	69
Pb–0.5%Co–7pass	1.1	0.6	82
Pb–0.5%Co–10pass	7.4	1.1	84

**Table 4:** Sub-grain size and dislocation density obtained by the Rietveld XRD analysis method.

Sample	Sub Grain Size (nm)	Dislocation Density (nm/nm^3^) * 10^−5^
Pb–10pass	1160	0.926
Pb–0.5%Ag–10pass	815	1.230
Pb–0.5%Co–10pass	174	5.673

## Data Availability

The raw/processed data required to reproduce these findings cannot be shared at this time as the data also form part of an ongoing study. If you need access to some of the raw data, please contact the corresponding author.

## Supplementary Materials

The following are available online at https://www.mdpi.com/article/10.3390/nano11051190/s1, Figure S1: Shear punch curves test for Pb–Ag, Pb–Co, and Pb at 10passes of ARB. Figure S2: Shear punch test of different samples. Figure S3: Tensile strengths and shear strengths of different specimens.
