# Supplementary material for: Enhanced Mechanical Properties and Microstructure of Accumulative Roll-Bonded Co/Pb Nanocomposite
Source: Nanomaterials (Basel). 2021 Apr 30;11(5):1190. doi: 10.3390/nano11051190 (PMC8147126; doi:10.3390/nano11051190)
Supplement: Supplementary file 1 [file nanomaterials-11-01190-s001.zip › nanomaterials-1191383-supplementary.pdf]

nanomaterials

Supporting Information

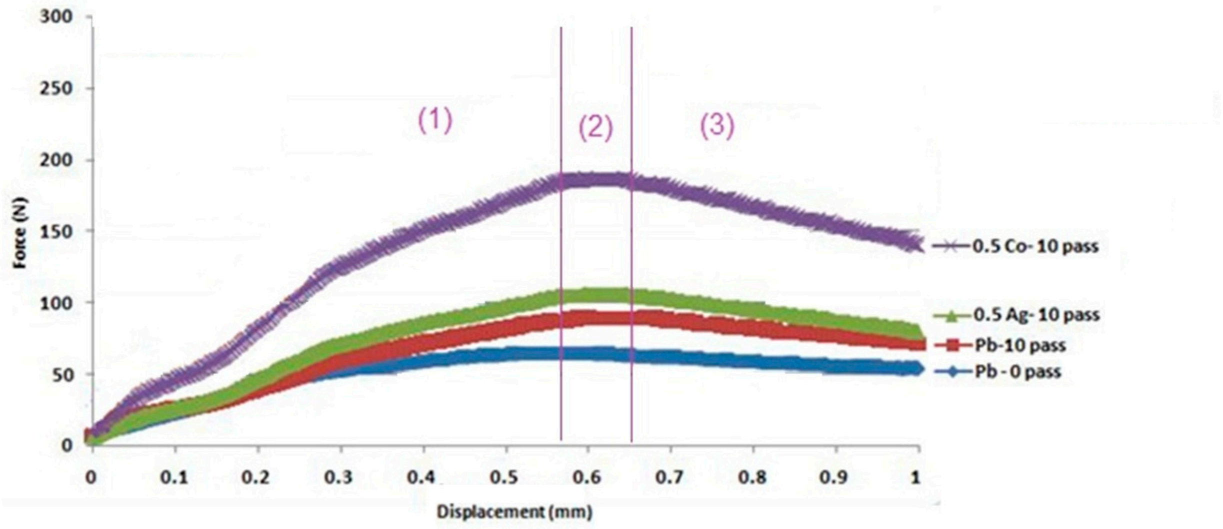

Figure S1. Shear punch curves test for Pb-Ag, Pb-Co and Pb at 10passes of ARB.

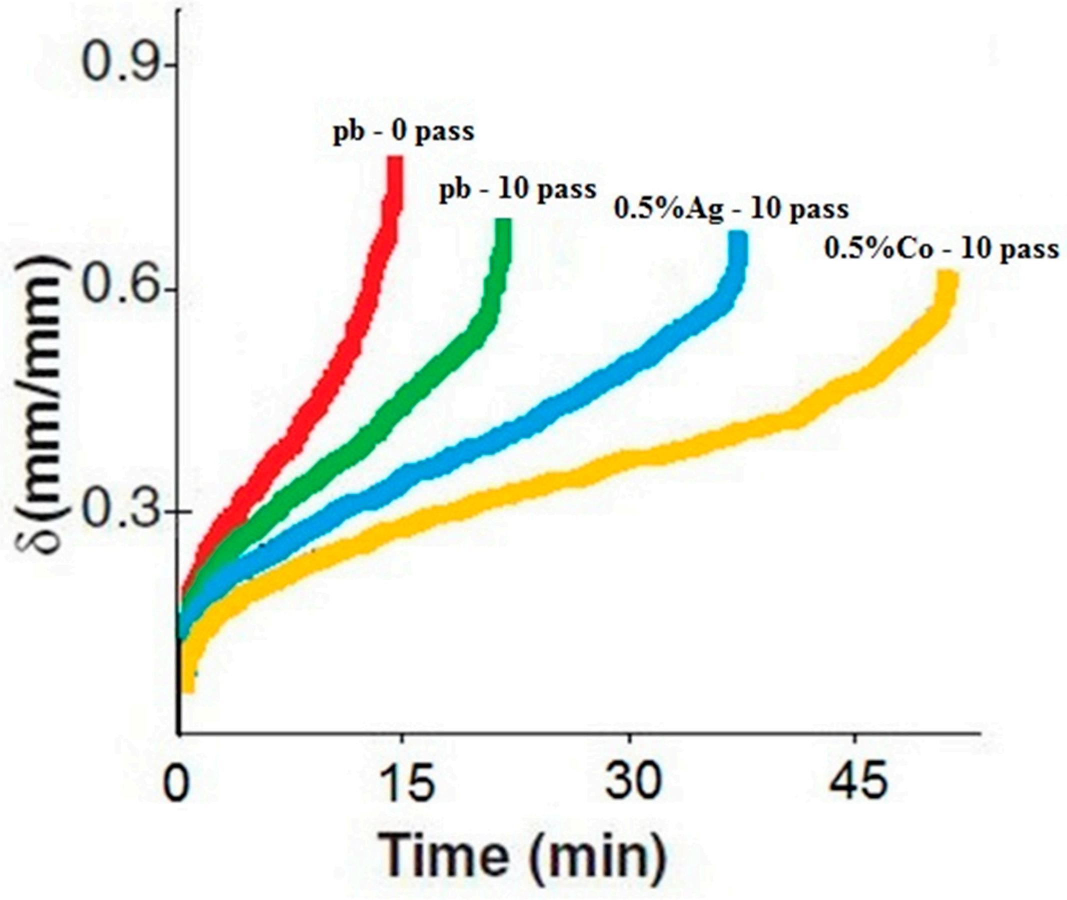

Figure S2. Shear punch test of different samples.

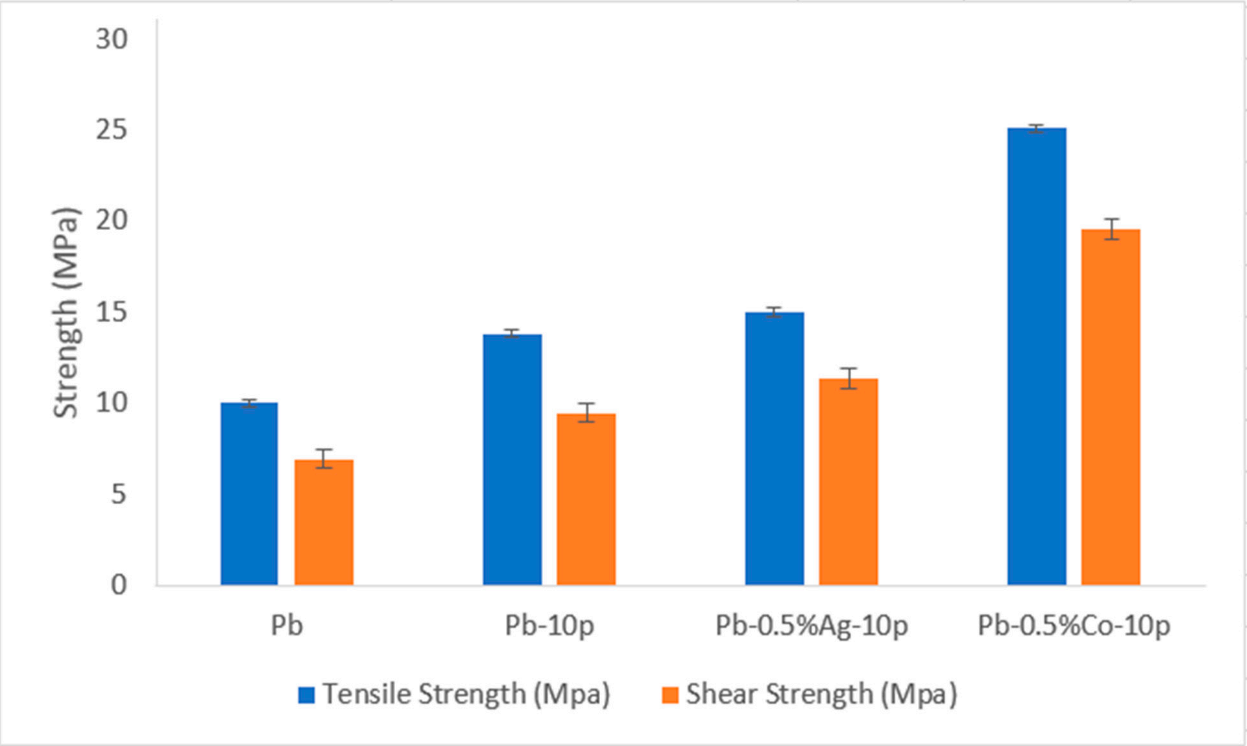

Figure S3. Tensile strengths and shear strengths of different specimens.
